# Hypoxia inducible factor-1α promotes trichogenic gene expression in human dermal papilla cells

**DOI:** 10.1038/s41598-023-28837-0

**Published:** 2023-01-27

**Authors:** Jieun Seo, Lei Yan, Tatsuto Kageyama, Ayaka Nanmo, Yang-Sook Chun, Junji Fukuda

**Affiliations:** 1 Faculty of Engineering, Yokohama National University, 79-5 Tokiwadai, Hodogaya-ku, Yokohama, Kanagawa 240-8501 Japan; 2 Kanagawa Institute of Industrial Science and Technology, 3-2-1 Sakado, Takatsu-ku, Kawasaki, Kanagawa 213-0012 Japan; 3 Department of Biomedical Sciences, Seoul National University College of Medicine, Seoul, 03080 Republic of Korea

**Keywords:** Cell biology, Physiology, Diseases, Engineering

## Abstract

Dermal papilla cells (DPCs) play critical roles in hair follicle development, but the underlying mechanisms that contribute to hair regeneration have yet to be fully elucidated, particularly in terms of alterations in androgenetic alopecia patients. In this study, we demonstrated that hypoxia-inducible factor-1α (HIF-1α) is suppressed in scalp tissues of androgenetic alopecia patients and potentially associated with hair follicle development. Using RT-qPCR and western blot, we found that mRNA and protein levels of trichogenic genes, LEF1 and versican (VCAN), were attenuated in HIF-1α knockdown DPCs. Under an in vivo mimicked environment in a three-dimensional spheroid culture, HIF-1α-suppressed DPCs downregulated the expression of hair induction-related genes. Finally, treatment with a HIF-1α activator resulted in the elevated expression of trichogenic genes in DPCs. This study highlights the importance of dermal HIF-1α expression in regulating trichogenic genes and provides a promising therapeutic target and a fundamental tissue engineering approach for hair loss treatment.

## Introduction

The hair follicle is unique to mammals and undergoes cyclic transformations for its entire lifetime^1^. The development and growth process of hair follicles is regulated by a distinct population of mesenchymal cells, dermal papilla cells (DPCs), which exist in the proximal end of the hair follicle^2^. Dermal signals such as Wnt signaling and Sonic hedgehog (Shh) signaling induce the formation of hair placodes and promote early DP development with maturation, respectively^3–5^. Thus, maintaining and elevating hair inductivity via dermal signals in DPCs could be an effective therapeutic strategy for recovering hair loss, but it is still poorly understood.

To clarify the molecular mechanisms in DPCs, many studies have utilized three-dimensional (3D) cell culture due to the maintenance of hair inductivity. Because conventional monolayer cell culture does not fully reflect niche effects within direct and reciprocal interactions between cells, three-dimensional cell culture is considered an alternative for mimicking the in vivo microenvironment. In DPCs, three-dimensional culture offers enhanced de novo human hair-follicular growth and restores the characteristics of DPCs, while three-dimensionally grown aggregates show internal necrosis with low oxygen permeability^6–8^. To overcome and adjust to the low oxygen environment, cells exhibit hypoxia-inducible factor-1 (HIF-1), the key transcription factor for oxygen sensing and oxygen-related signal transduction^9^. Over the decades, HIF-1-mediated cellular responses have been well studied, especially in cancer. HIF-1 governs the transactivation of genes related to proliferation, survival, invasion, angiogenesis, and metabolism; thus, tumoral HIF-1 is considered a promising target for treating cancer^10,11^. However, the relevance of HIF-1 in hair physiology remains elusive, although evidence supporting its role in the process exists.

In DPCs, hypoxia exposure resulted in an increase in proliferation by modulating lactate dehydrogenase activity^12^. Along with the growth of DPCs, hypoxia also stimulates hair inductivity via paracrine effects that include secretion of growth factors and hypoxia-induced reactive oxygen species (ROS) generation^13^. The importance of hypoxia in the hair inductivity of DPCs is well described by the above studies, while the relevance of HIF-1α in the process has not yet been examined. Our previous study showed that human DPCs embedded in collagen microgels exhibit significantly high de novo hair regeneration activity when transplanted with epithelial cells into the back skin of nude mice^14,15^. The elevated trichogenic ability was found to be closely associated with the PI3K-Akt signaling pathway^16^. More recently, we reported an approach to reconstruct matured hair follicles in vitro from dissociated single cells, in which PI3K/AKT signaling pathway also played an important role^17^. PI3K-Akt signaling pathway is a well-known pathway for the translation of HIF-1α^18^. Furthermore, trichogenic DP cell markers, including lymphoid enhancer binding factor 1 (LEF1) and versican (VCAN), are known to be regulated by HIF-1α in embryonic stem (ES) cells and endothelial cells, respectively^19–22^. Considering these emerging evidences related to HIF-1α-mediated hair regeneration, we hypothesized that HIF-1α could modulate trichogenic gene expression in DPCs. Herein, we showed that HIF1A was downregulated in androgenetic alopecia (AGA) tissues and that HIF1A expression levels were positively correlated with hair inductivity-related genes by using informatic analysis. Next, the involvement of HIF-1α in the regulation of trichogenic genes was demonstrated with HIF-1α knockdown (KD) DPCs. Furthermore, gene chip analysis showed the possibility of upregulation of HIF-1α in DPC spheroids; thus, the trichogenic gene levels were checked with DPC spheroids. Under the environment with endogenously upregulated HIF-1α levels, HIF-1α silencing also resulted in the suppression of trichogenic gene levels. Finally, treatment with a HIF-1α activator enhanced the hair inductive potential of DPCs, indicating that HIF-1α is a promising therapeutic target for treating hair loss.

## Results

### The expression level of HIF1A is positively correlated with hair function-related genes in human scalp tissues

**Figure 1:**
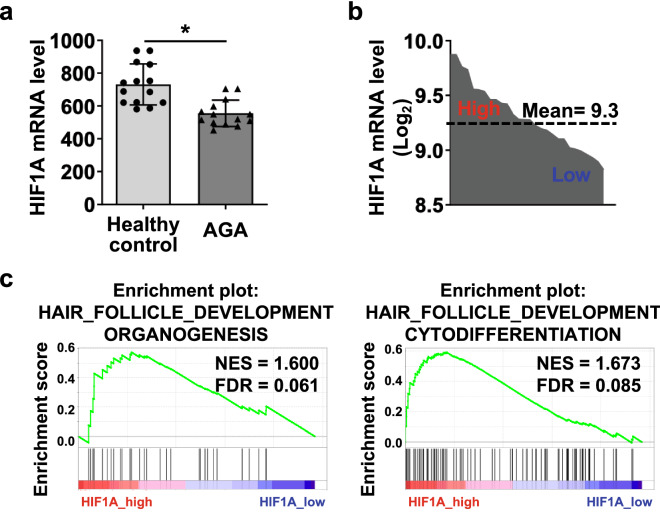
HIF1A is highly expressed in healthy scalp tissues, and its levels are positively correlated with hair function-related genes. **(a)** Linear HIF1A mRNA expression levels in healthy scalp tissues (healthy control, *n* = 14) and androgenetic alopecia scalp tissues (AGA, *n* = 14), which were collected from the GEO database (GSE90594). The results represent the means and standard error of the mean (SEM). *P < 0.05. **(b)** High- and low-HIF1A groups were categorized based on the mean value of log_2_ transformed HIF1A mRNA level (high, n = 12; low, n = 16, mean = 9.3). **(c)** Representative gene set enrichment analysis (GSEA) plots of the high- and low-HIF1A groups.

To investigate the role of HIF-1α in hair growth and development, we analyzed HIF1A mRNA levels using the NCBI GEO database (GSE90594). In scalp tissues with androgenetic alopecia, which show progressive hair loss, HIF1A mRNA levels were significantly repressed compared to those in tissues from healthy individuals (Fig. 1a). Next, we divided the samples into HIF1A-high and HIF1A-low groups (Fig. 1b) and conducted gene set enrichment analysis (GSEA) to demonstrate the relationship between HIF1A and hair regeneration-related genes. The results showed that hair growth- and hair follicle development-related genes were enriched in HIF1A-high samples (Fig. 1c, Fig. S1a,b). Taken together, these data indicate that HIF1A may be involved in hair growth and regeneration.

### Knockdown of HIF-1α attenuates trichogenic gene expression in dermal papilla cells

**Figure 2:**
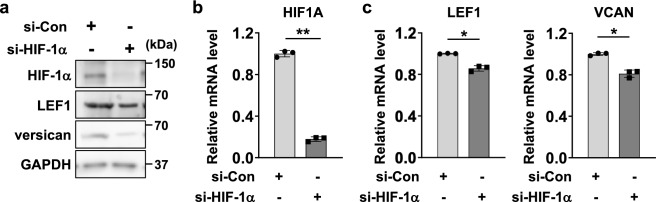
HIF-1α knockdown suppresses mRNA levels of trichogenic genes in dermal papilla cells (DPCs). **(a,b)** DPCs were treated with control siRNA (si-Con) or HIF-1α (si-HIF-1α) for 48 h. Cells were subjected to western blotting (**a**) with HIF-1α and GAPDH antibodies or real-time quantitative PCR (RT‒qPCR, **b**). **(c)** si-Con- or si-HIF-1α-transfected DPCs were used to perform RT–qPCR to determine the mRNA levels of genes involved in trichogenesis (LEF1 and VCAN). Relative mRNA levels are presented as the means and standard deviation (SD) calculated with data from 3 independent experiments.

As HIF-1α is responsible for hair function in human scalp tissues, we examined HIF-1α-mediated regulation of trichogenic genes in dermal papilla cells (DPCs). We first confirmed the knockdown of HIF-1α in DPCs (Fig. 2a,b). After checking the efficiency of HIF-1α knockdown in DPCs, we further identified HIF-1α-dependent trichogenic gene regulation in DPCs. As LEF1 and versican (VCAN) are known as HIF-1α target genes, we assessed the LEF1 and versican (VCAN) expression levels in HIF-1α-silenced DPCs. Both protein and mRNA expression levels of LEF1 and versican (VCAN) were suppressed in si-HIF-1α-treated DPCs compared to the expression levels observed in control DPCs (Fig. 2a,c). Meanwhile, treatment of si-HIF-1α did not significantly affect other trichogenic genes including alkaline phosphatase (ALP) or WNT5A (Fig. S2a). Collectively, knockdown of HIF-1α drives the suppression of trichogenic gene levels in DPCs.

### Three-dimensionally cultured DPCs show upregulated HIF-1α, and HIF-1α silencing represses trichogenic gene expression

**Figure 3:**
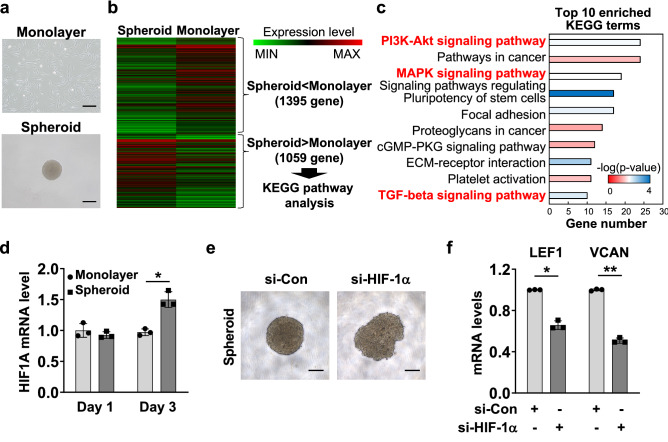
Three-dimensional (3D) culture of DPCs shows high expression of HIF1A, and HIF-1α regulates trichogenic genes in DPC spheroids. **(a)** Representative images of monolayer cultured or three-dimensionally cultured DPCs. **(b)** Gene chip analysis was conducted with monolayer cultured DPCs and DPC spheroids. A total of 1059 genes (upregulated in DPC spheroids compared to monolayer cultured DPCs, threshold of Log_2_FC > 1) were selected for Kyoto Encyclopedia of Genes and Genomes (KEGG) pathway analysis. **(c)** The list of top enriched KEGG pathways. **(d)** Total RNA from monolayer cultured DPCs or DPC spheroids was extracted and then subjected to RT‒qPCR to determine HIF1A mRNA levels (mean ± SD, n = 3). **(e,f)** DPCs were treated with si-Con or si-HIF-1α for 48 h and subsequently utilized for spheroid organization (**e**). LEF1 and VCAN mRNA levels were measured using RT‒qPCR (**f**). The data are presented as the means and SD calculated with data from 3 independent experiments.

We next profiled the expression pattern of monolayer cultured DPCs (2D) and DPC spheroids (3D) using gene chip analysis. We selected 1059 genes that were elevated in DPC spheroids compared to monolayer culture DPCs for KEGG pathway analysis (Fig. 3a,b). Among the top 10 enriched KEGG pathways, pathways related to HIF-1α upregulation included the PI3K-Akt signaling pathway, MAPK signaling pathway, and TGF-beta signaling (Fig. 3c). Furthermore, HIF1A mRNA levels in DPC spheroids were increased significantly compared to monolayer cultured DPCs (Fig. 3d). Thus, we further confirmed the role of HIF-1α in DPC spheroids. After treatment with si-HIF-1α in DPCs, spheroids were structured and collected to verify the alteration of trichogenic gene levels (Fig. 3e). In si-HIF-1α-treated DPC spheroids, the mRNA levels of trichogenic genes, including LEF1 and VCAN, were significantly repressed compared to si-control-treated DPC spheroids (Fig. 3f). Since cellular responses to stimuli is known to be improved with 3D culture (compared to 2D culture), the differences in expression of both genes caused by the HIF-1α knockdown are more than double in the cells in 3D culture than those in 2D culture (Fig. 2c)^23^. Collectively, these data indicate that HIF1A is highly expressed in DPC spheroids and positively correlated with the expression of trichogenic genes.

### HIF-1α activator promotes trichogenic gene expression in DPCs

**Figure 4:**
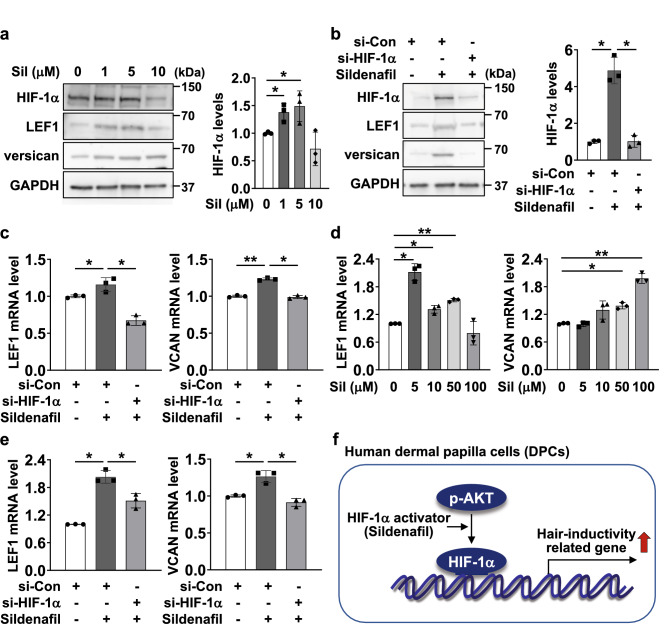
The HIF-1α activator sildenafil activates trichogenic gene expression in DPCs. **(a)** DPCs were incubated with sildenafil (1, 5, 10 mM) for 24 h and subjected to western blotting. Quantification was performed based on GAPDH expression levels by using ImageJ. **(b,c)** Cells were transfected with si-Con or si-HIF-1α and then treated with 5 mM sildenafil for 24 h. Cell lysates were subjected to western blotting (following quantification of the blots, **b**) or RT‒qPCR (**c**). **(d)** DPC spheroids were incubated with sildenafil (5, 10, 50, 100 mM) for 24 h and lysed for RT‒qPCR to determine LEF1 and VCAN mRNA levels. **(e)** si-Con- or si-HIF-1α-transfected DPCs were utilized for 3D culture. The formed spheroids were lysed for RNA extraction, and RT‒qPCR was performed. **(f)** The proposed mechanism by which HIF-1α accelerates trichogenic gene expression levels in DPCs. The data are presented as the means and SD calculated with data from 3 independent experiments.

As a HIF-1α activator, sildenafil impacts HIF-1α translational upregulation^24^, and we verified whether sildenafil treatment could elevate trichogenic gene levels in DPCs. Treatment with sildenafil resulted in an increase in the endogenous protein levels of HIF-1α, LEF1, and versican in a dose-dependent manner (Fig. 4a, Fig. S3a). We also confirmed that sildenafil-mediated upregulation of HIF-1α, LEF1, and versican were inhibited in si-HIF-1α-treated DPCs (Fig. 4b, Fig. S3b). We then explored the effect of sildenafil on the regulation of trichogenic gene levels in DPCs. Interestingly, sildenafil elevated LEF1 and VCAN mRNA levels, while HIF-1α knockdown suppressed sildenafil-induced trichogenic gene expression (Fig. 4c). To further verify this phenomenon in 3D culture, the enhanced LEF1 and VCAN mRNA levels were checked with sildenafil-treated DPC spheroids (Fig. 4d). Finally, we demonstrated that sildenafil-induced LEF1 and VCAN mRNA levels are HIF-1α dependent using DPC spheroids in which HIF-1α was silenced. Sildenafil accelerated the mRNA expression of LEF1 and VCAN, while this effect was abrogated in HIF-1α KD DPC spheroids (Fig. 4e). These results revealed that sildenafil-induced trichogenic gene expression is dependent on HIF-1α levels in DPCs (Fig. 4f).

## Discussion

HIF-1α is a key transcription factor governing diverse physiological processes through transactivating genes, including cell survival, migration, metabolism, and angiogenesis. We have recently revealed an association of hair-inducing functions in DPCs with the PI3K-Akt signaling pathway, which is considered one of the pathways for upregulating HIF-1α translation^16,25,26^. Both this study and our previous study reported that intracellular gene levels are regulated by the transcription factor, suggesting that targeting HIF-1α could be an effective strategy for reversing hair loss. In this study, we analyzed the downregulated HIF1A mRNA levels in scalp tissues of AGA patients, which implies that the suppression of HIF-1α levels is positively correlated with hair thinning and loss^27^. Moreover, we verified that HIF-1α silencing repressed trichogenic gene levels in DPCs. Since DPCs are the main cells within hair follicles that regulate hair growth by exhibiting dermal signals, discovering mechanisms in DPCs is reliable for determining the molecular targets to treat AGA and hair regenerative medicine^3–5^. Even though we found that HIF-1α regulated trichogenic gene expression in DPCs in a dose-dependent manner, further analysis based on HIF-1α chromatin immunoprecipitation should reveal direct target genes of HIF-1α in DPCs, which is an open question to date.

Next, we demonstrated the role of HIF-1α in the regulation of hair inductivity-related genes in three-dimensionally cultured DPCs (DPC spheroids). Conventional monolayer culture (2D culture) of DPCs is reported to lose their trichogenic ability with passage^6,7^. Thus, 3D culture has been introduced to restore the physiological characteristics of DPCs in vitro by mimicking three-dimensional cell‒cell communications in the dermal microenvironment^3,28,29^. Using the gene chip assay, the predominant signaling pathways in DPC spheroids (compared to 2D culture) were identified, including the PI3K-Akt signaling pathway, MAPK signaling pathway, and TGF-beta signaling pathway, which are involved in the induction of HIF-1α translation^25,26^. Our previous study with human DPCs elucidated the importance of PI3K/Akt signaling pathway in regulating the trichogenic ability. DPCs embedded in collagen microgels three-dimensionally showed high hair regeneration activity, which was suppressed by the treatment of PI3K-Akt inhibitor, LY294002^16^. Not only DPC spheroids but also matured hair follicles with mouse-derived epithelial cells and mesenchymal, in vitro hair follicle sprouting was inhibited by the treatment of LY294002, indicating PI3K-Akt signaling pathway is critical for hair follicle morphogenesis^17^. Considering our previous studies and translational mechanisms of HIF-1α, the enhancement of trichogenic gene levels might be regulated by HIF-1α. Notably, spheroids are grown in three dimensions, and an intrahypoxic region is created as an indispensable region due to poor oxygen permeability as a solid tumor. In human solid tumors, the intrahypoxia region accounts for 19% to 70% of the tumor volume; thus, adaptation to hypoxia via the induction of HIF-1α is essential for cell survival and function^30,31^. Therefore, the upregulation of HIF-1α is a reasonable consequence in forming DPC spheroids, but the result might imply a positive correlation between HIF-1α and hair inductivity. We revealed that HIF-1α-KD DPC spheroids exhibited lower expression of hair inductivity-related genes, indicating that HIF-1α is responsible for regulating hair inductivity in DPC spheroids. Finally, we further demonstrated that sildenafil, which is also known as a HIF-1α activator, could accelerate trichogenic gene expression levels in DPCs and DPC spheroids. Previously, Choi et al. revealed that sildenafil increases hair growth in C57BL/6 mice, but the detailed mechanisms were unknown^32^. a Furthermore, hypoxic conditions are known to elevate DP cell proliferation and hair inductivity, but they are not suitable for long-term cell culture because prolonged hypoxia can induce cell death^12,13,33^. Based on our results, treating sildenafil could be a convenient method for gathering DPCs with high hair inductivity by activating HIF-1α, not incubating cells under hypoxia.

In conclusion, we demonstrate here that HIF-1α drives trichogenic gene expression levels in DPCs. Maintaining DPCs with high HIF-1α levels could be applicable to prepare DPCs with enhanced hair inductivity for hair implantation. From a therapeutic perspective, targeting HIF-1α can be a potential strategy for recovering hair loss.

## Methods

### Monolayer and three-dimensional cell culture

Human dermal papilla cells (DPCs) were obtained from PromoCell (Heidelberg, Germany). DPCs were cultured with follicle dermal papilla cell growth medium (DPCGM; PromoCell, Heidelberg, Germany). Incubator gas tension was maintained at 21% O_2_ and 5% CO_2_ at 37 °C. DPCs at passages 3 to 5 were used in the in vitro studies. For three-dimensional cell culture, DPCs (1 × 10^4^ cells/well) were suspended in the culture medium and seeded onto nonadhesive 96-well plates (PrimeSurface Plate 96U, Sumitomo Bakelite Co., Japan). The formed spheroids were collected after brief washing with PBS and then lysed for RNA extraction.

### Chemicals and short interfering RNAs (siRNAs)

The HIF-1α activator sildenafil was purchased from Sigma‒Aldrich (St. Louis, MO, USA). The sequences targeting HIF-1α and the control were synthesized by Integrated DNA Technologies Japan (Tokyo, Japan). The design of si-HIF-1α was as follows: 5′-GGGAUUAACUCAGUUUGAACUAACT-3′ and 3′-UACCCUAAUUGAGUCAAACUUGAUUGA-5′.

### Informatics analysis

The scalp gene expression in patients with androgenetic alopecia (AGA) was assessed with the NCBI Gene Expression Omnibus (GEO) dataset (https://www.ncbi.nlm.nih.gov/geo, GSE90594). HIF1A mRNA expression levels (A_24_P56388 at probe) were evaluated between the healthy controls (n = 14) and AGA patients (n = 14). For gene set enrichment analysis (GSEA; https://www.gsea-msigdb.org/gsea/index.jsp), the mean value of A_24_P56388 (corresponding to HIF1A) was used as the criterion for categorizing the low- and high-HIF1A expression groups. A false discovery rate (FDR) less than 0.25 was considered statistically significant.

### Western blotting

For protein extraction, 2D cultured cells were washed with PBS and sequentially lysed with RIPA lysis buffer (EzRIPA Lysis kit; ATTO, Tokyo, Japan). The cell lysates were incubated at 4 °C for 15 min and centrifuged for 10 min at 14,000×*g*. The supernatant was collected and mixed with 2× sodium dodecyl sulfate (SDS) sample buffer. The proteins were separated on SDS‒PAGE gels (Bio-Rad, Hercules, CA, USA) and transferred onto Immobilon-P membranes (Merck KGaA, Darmstadt, Germany). The membranes were blocked with 3% BSA in Tris-buffered saline containing 0.1% Tween-20 (TBST) solution for 1 h at room temperature. The blots were cut with the adequate size for inhibiting non-specific detection and incubated with primary antibodies (HIF-1α, 1:1000 dilution, Abcam; GAPDH, 1:2000 dilution, Cell Signaling Technology) at 4 °C overnight. The membranes were washed three times with TBST solution, incubated with a horseradish peroxidase-conjugated secondary antibody (1:2000 dilution, Cell Signaling Technology) for 1 h and visualized using chemiluminescent reagents (ECL Prime; GE Healthcare, Buckinghamshire, UK) and an Amersham imager 600 RGB (Cytiva, Tokyo, Japan). The relative HIF-1α expression levels were calculated using GAPDH as a reference protein.

### Real-time quantitative reverse transcription polymerase chain reaction

Total RNA from spheroids or cultured cells was extracted using the RNeasy Mini Kit (Qiagen, Hilden, Germany), and cDNA synthesis was performed in a ReverTra Ace qPCR RT kit (TOYOBO, Japan). Quantitative real-time PCR (qRT‒PCR) on 96-well optical plates was performed using QuantStudio 3 (Thermo Fisher Scientific) with TB Green Premix EX Taq II (Takara Bio, Shiga, Japan). The sequences for qRT‒PCR primers were designed as follows: VCAN (5′-GGCACAAATTCCAAGGGCAG-3′, 3′-TCATGGCCCACACGATTAACA-5′), LEF1 (5′-CCCGATGACGGAAAGCAT-3′, 3′-TCGAGTAGGAGGGTCCCTTGT-5′), HIF1A (5′-TGCAGAATGCTCAGAGAAAGCGAA-3′, 3′-GCTGCATGATCGTCTGGCTGCT-5′), and GAPDH (5′-GCACCGTCAAGGCTGAGAAC-3′, 3′-TGGTGAAGACGCCAGTGGA-5′). The mRNA values of targeted genes were normalized to the GAPDH expression level.

### Gene chip analysis

Total RNA was extracted from DPCs in 2D and spheroid culture at 3 d using the RNeasy Mini Kit (Qiagen) according to the manufacturer’s instructions. Gene expression profiling was performed using GeneChip Human Genome U133 Plus 2.0 arrays (Thermo Fisher Scientific). KEGG analysis was performed using the R package clusterProfiler. The upregulated genes of DPCs in spheroid culture compared to 2D culture were selected with the threshold of Log_2_FC > 1. The significantly upregulated genes in DPC spheroids were used for Kyoto Encyclopedia of Genes and Genomes (KEGG) pathway analysis with the Database for Annotation, Visualization, and Integrated Discovery (http://david.abcc.ncifcrf.gov/). The top 10 enriched pathways were identified with upregulated and differentially expressed genes (DEGs).

### Statistical analysis

All generated data were expressed as the means and standard deviation or standard error of the mean. Two-tailed, paired Student’s *t* test was conducted to compare differences between the groups using GraphPad Prism 8 software. Differences were considered statistically significant when *P* < 0.05.

## Supplementary Information

Supplementary Figures.

Supplementary Information.

## Data Availability

All data used and analyzed during the study are included in the manuscript.
