# Supplementary material for: Hypoxia inducible factor-1α promotes trichogenic gene expression in human dermal papilla cells
Source: Sci Rep. 2023 Jan 27;13:1478. doi: 10.1038/s41598-023-28837-0 (PMC9883512; doi:10.1038/s41598-023-28837-0)
Supplement: Supplementary file 1 — Supplementary Figures. [file 41598_2023_28837_MOESM1_ESM.pdf]

Supplement figure 1

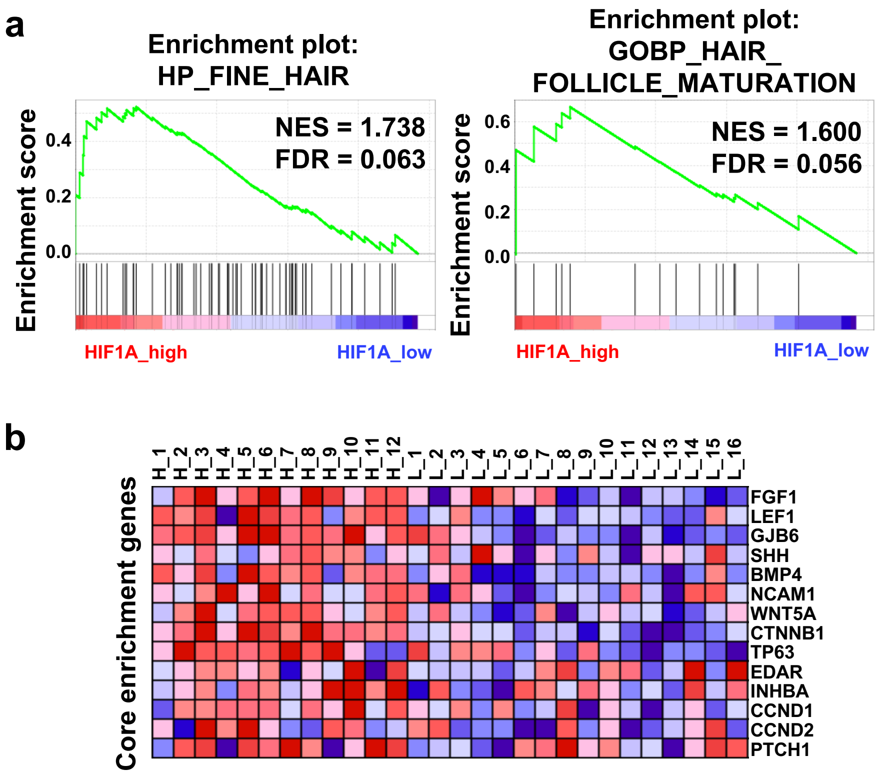

Supplemental Figure 1. The detailed information for informatic analysis shown in Figure 1.

(a) GSEA (Gene Set Enrichment Analysis) plots for the HP_FINE_HAIR and GOBP_HAIR_FOLLICLE_MATURATION gene sets in the high- and low-HIF1A groups. **(b)** Core enrichment genes in HAIR_FOLLICLE_DEVELOPMENT_ORGANOGENESIS based on the rank metric score > 0.3 were listed using GSEA (www.gsea-msigdb.org/gsea/index.jsp).

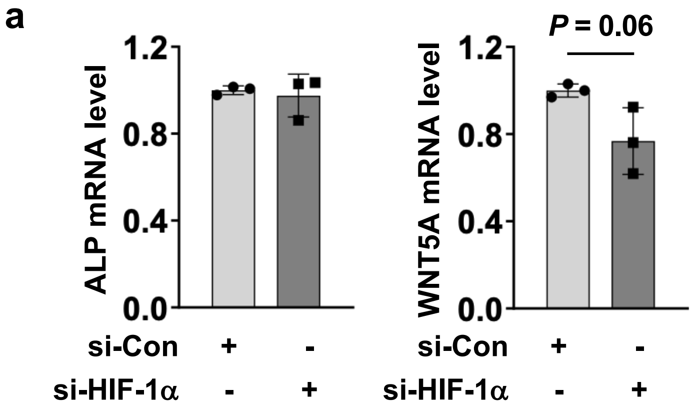

Supplemental Figure 2. mRNA levels of ALP and WNT5A in HIF-1α knockdown DPCs.

(a) DPCs were transfected with si-Con or si-HIF-1α for 48 h. Cell lysates were subjected to RT-qPCR to determine ALP and WNT5A mRNA levels (mean ± SD, n = 3).

Supplement figure 3

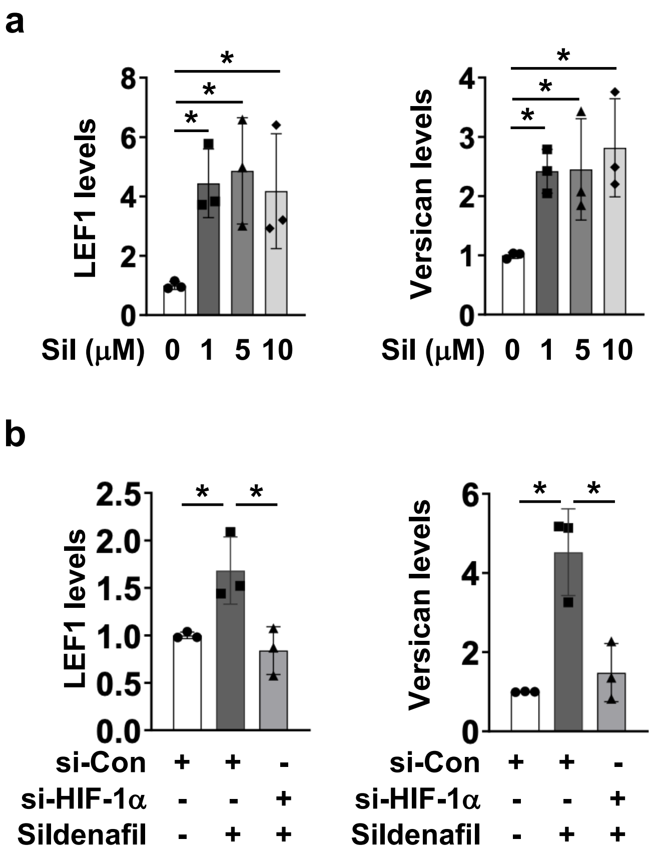

Supplemental Figure 3. The quantification data for western blotting shown in Figure 4.

(a) Silednafil (1, 5, 10 mM) treated DPCs were subjected to western blotting and the quantification was performed based on GAPDH protein levels by using ImageJ (main Fig. 4a, mean ± SD, n = 3). **(b)** si-Con or si-HIF-1α transfected cells were treated with 5 mM sildenafil, and then subjected to western blotting and the quantification (main Fig. 4b, mean ± SD, n = 3)
