# Supplementary material for: Hypoxia inducible factor-1α promotes trichogenic gene expression in human dermal papilla cells
Source: Sci Rep. 2023 Jan 27;13:1478. doi: 10.1038/s41598-023-28837-0 (PMC9883512; doi:10.1038/s41598-023-28837-0)
Supplement: Supplementary file 2 — Supplementary Information. [file 41598_2023_28837_MOESM2_ESM.pdf]

Fig. 2a

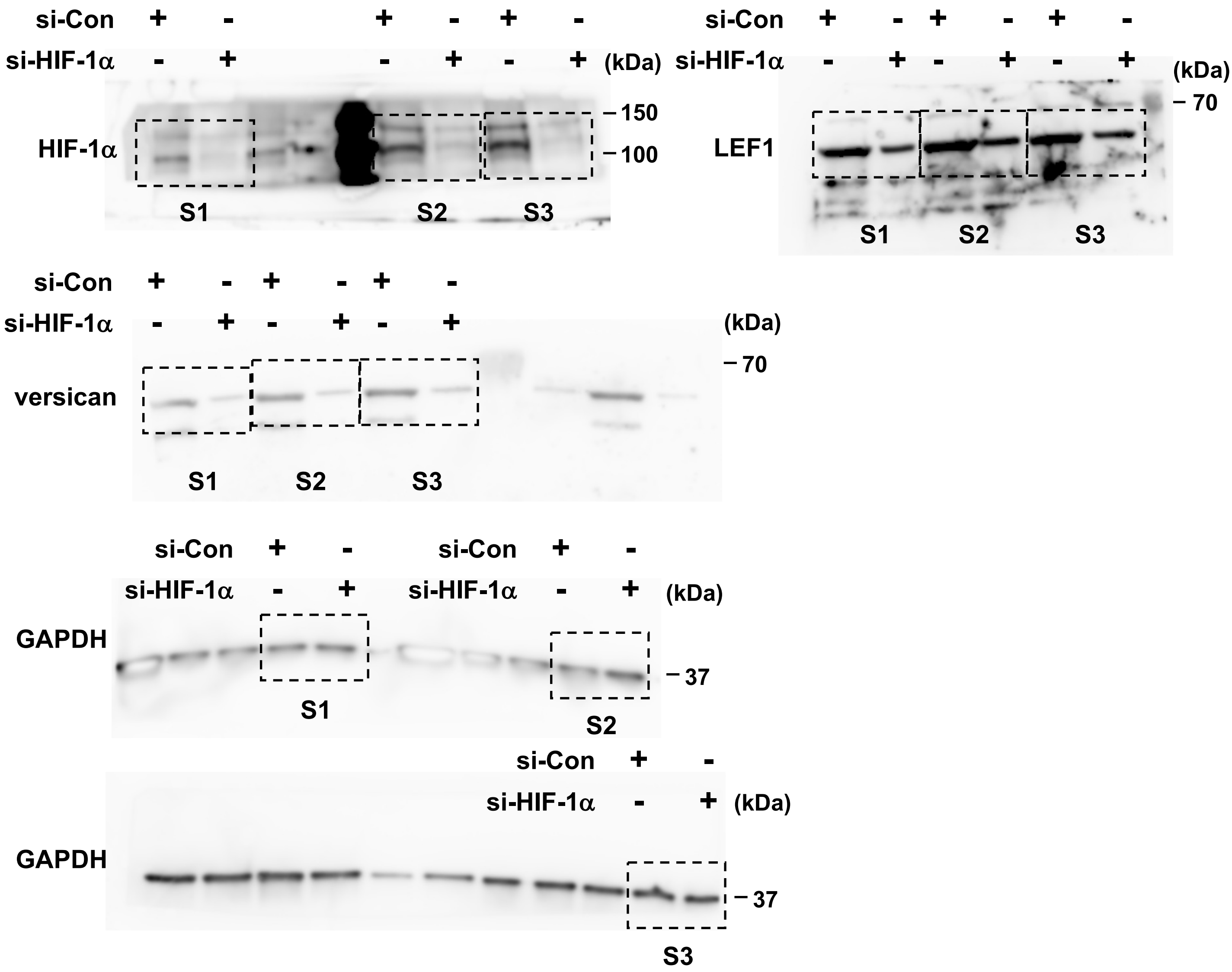
si-Con
si-HIF-1α
(kDa)
HIF-1α
150
100
S1
S2
S3
LEF1
70
versican
GAPDH
37

Fig. 4a

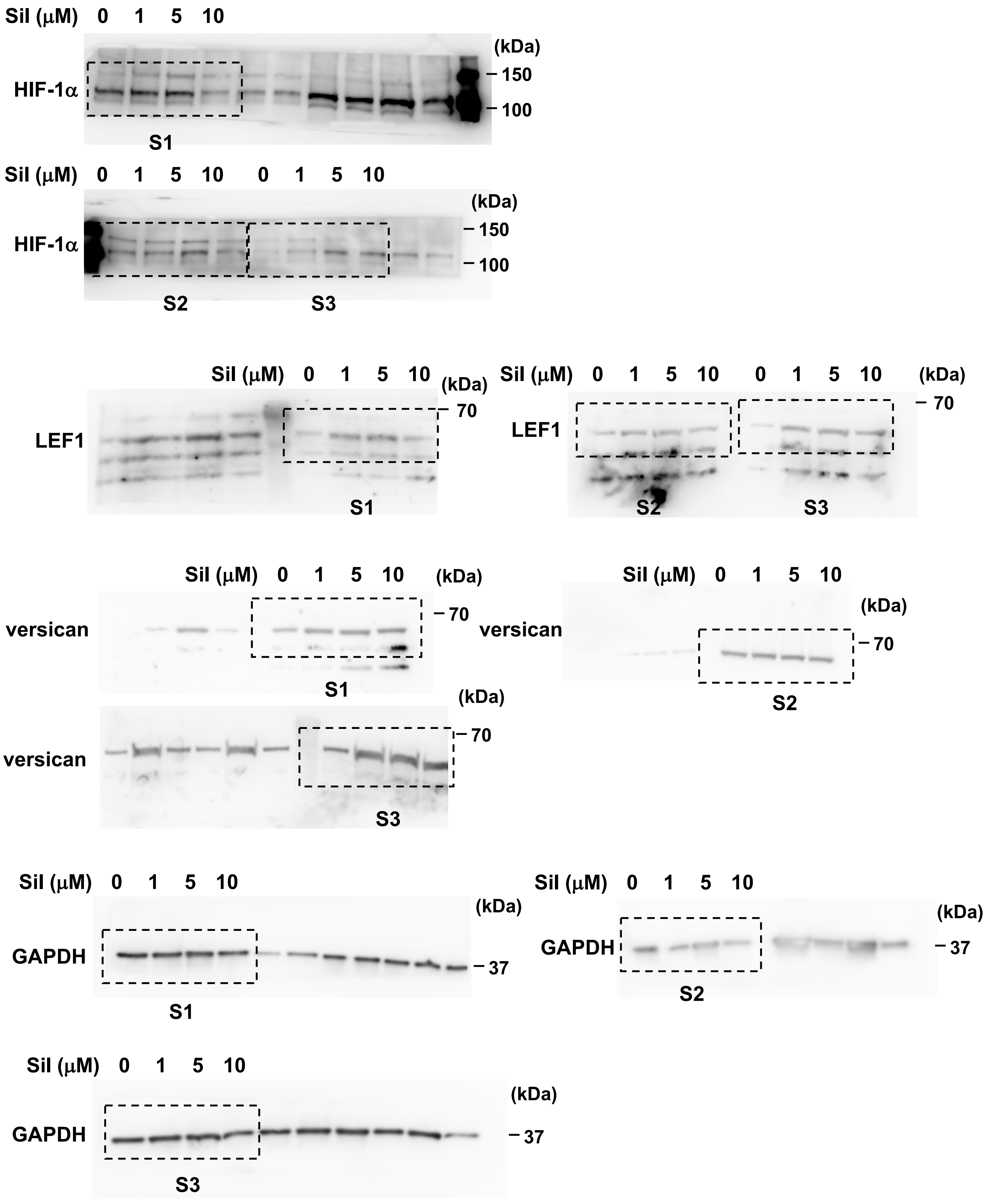
Sil (μM) 0 1 5 10
(kDa)
HIF-1α
150
100
S1
Sil (μM) 0 1 5 10 0 1 5 10
(kDa)
HIF-1α
150
100
S2
S3
Sil (μM) 0 1 5 10
(kDa)
LEF1
70
S1
Sil (μM) 0 1 5 10 0 1 5 10
(kDa)
LEF1
70
S2
S3
Sil (μM) 0 1 5 10
(kDa)
versican
70
S1
Sil (μM) 0 1 5 10
(kDa)
versican
70
S2
(kDa)
versican
70
S3
Sil (μM) 0 1 5 10
(kDa)
GAPDH
37
S1
Sil (μM) 0 1 5 10
(kDa)
GAPDH
37
S2
Sil (μM) 0 1 5 10
(kDa)
GAPDH
37
S3

Fig. 4b

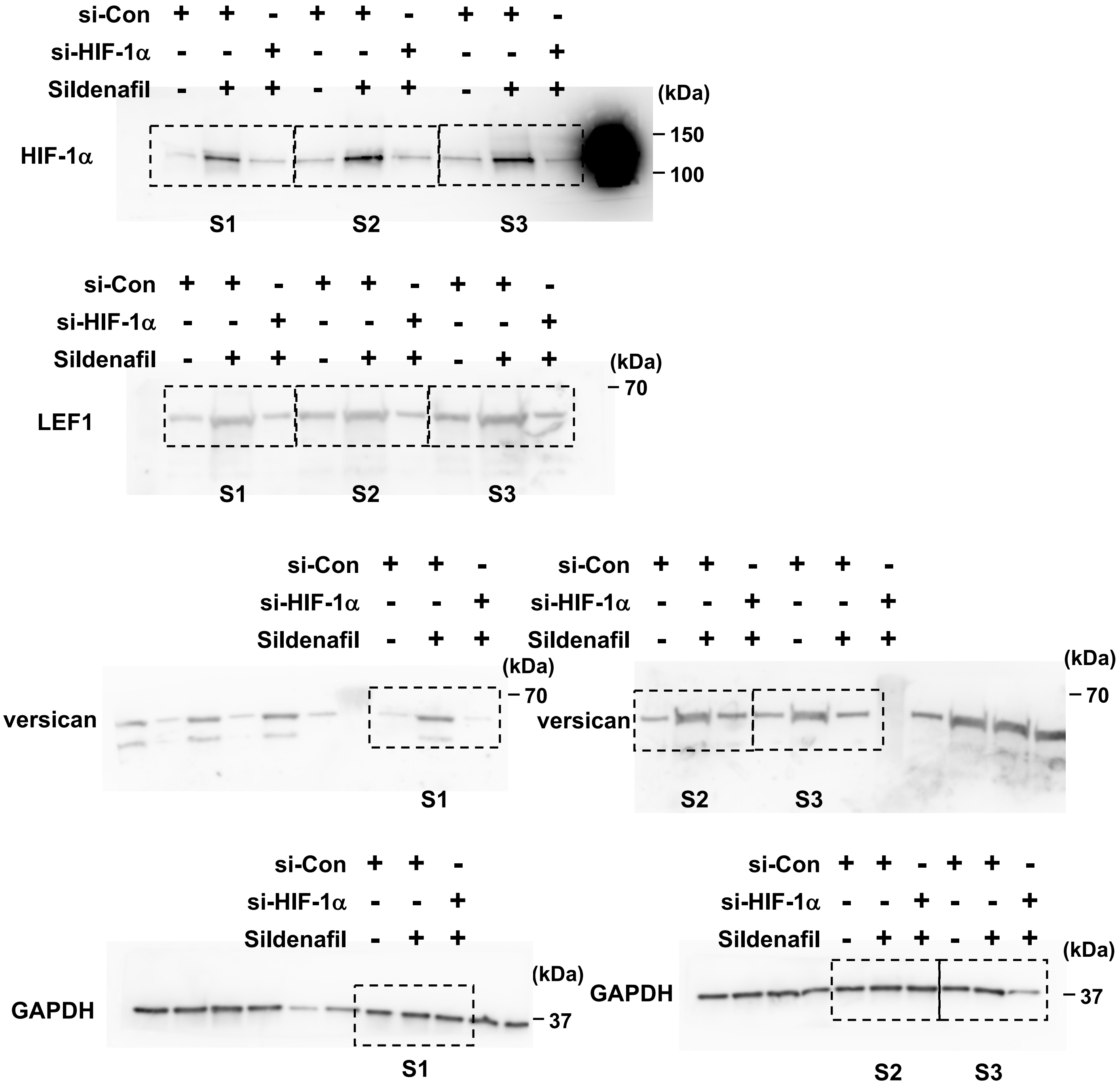
si-Con
si-HIF-1α
Sildenafil
(kDa)
HIF-1α
150
100
S1
S2
S3
LEF1
70
versican
GAPDH
37
